# Effects of medium-chain fatty acid supplementation levels in early nursery diets on growth performance, fecal score, gut permeability, energy status, and apparent total tract digestibility of weaning pigs

**DOI:** 10.1093/tas/txaf109

**Published:** 2025-08-13

**Authors:** Jannell A Torres, Madison J Mejia, Chan Ho Kwon, Eva S Safaie, Ellen Davis, Michaela P Metz, Young Dal Jang

**Affiliations:** Department of Animal and Dairy Science, University of Georgia, Athens, GA 30602, USA; Department of Animal and Dairy Science, University of Georgia, Athens, GA 30602, USA; Department of Animal and Dairy Science, University of Georgia, Athens, GA 30602, USA; Department of Animal and Dairy Science, University of Georgia, Athens, GA 30602, USA; Fortiva, a division of Land O’Lakes, Arden Hills, MN 55126, USA; Fortiva, a division of Land O’Lakes, Arden Hills, MN 55126, USA; Department of Animal and Dairy Science, University of Georgia, Athens, GA 30602, USA

**Keywords:** β-hydroxybutyrate, growth performance, gut permeability, medium-chain fatty acid, nutrient digestibility, weaning pigs

## Abstract

This study was conducted to evaluate the effects of dietary medium-chain fatty acid (**MCFA**) levels on growth performance, fecal score, gut permeability, energy status, and apparent total tract digestibility (**ATTD**) of weaning pigs. At weaning, a total of 100 pigs [18.4 ± 1.8 d of age; initial body weight (**BW**) of 5.72 ± 1.4 kg] were allotted to 5 treatments in 5 replicates with 4 pigs per pen for a 35-d feeding trial. Treatments were dietary MCFA levels of 0.0, 0.2, 0.5, 1.0, and 1.5% for d 0 to 21 postweaning (Phase 1) replacing soybean oil and a common nursery diet was fed to all pigs for d 21 to 35 postweaning (Phase 2). With increasing dietary MCFA levels, quadratic increases were observed in BW (*P* ≤ 0.05), average daily gain (**ADG**; *P* ≤ 0.05), and average daily feed intake (**ADFI**; *P* = 0.08, tendency) in d 0 to 14 postweaning, peaking at the 0.5% MCFA level. The BW from d 21 to 35 postweaning and ADG in d 0 to 21 and 21 to 35 postweaning increased linearly with increasing MCFA levels (*P* ≤ 0.05), while linear and quadratic increases were observed in overall ADG (*P* ≤ 0.05), with the greatest value observed at the 1.0% MCFA level. The ADFI increased linearly (*P* ≤ 0.05) with increasing MCFA levels in d 21 to 35 postweaning and overall period, while gain-to-feed ratio was not different in each phase and overall period. Fecal score was not affected in the overall period (*P* > 0.21). The ATTD of gross energy (*P* ≤ 0.05), dry matter (*P* = 0.10, tendency) and crude protein (*P* = 0.07, tendency) increased linearly with increasing MCFA levels. At d 7 postweaning, plasma β-hydroxybutyrate levels decreased linearly (*P* = 0.06, tendency) with increasing MCFA levels, while plasma free fatty acid levels showed a quadratic response (*P* = 0.06, tendency) with the lowest levels at the 0.5% MCFA level. Increasing MCFA levels decreased plasma diamine oxidase levels at d 7 postweaning (*P* = 0.06, tendency), while showing quadratic decreases in plasma d-lactate levels at d 7 (*P* ≤ 0.05) and 21 (*P* = 0.10, tendency) postweaning, with lower values observed at the 0.2% to 1.0% MCFA levels compared to the 0.0% MCFA level. In conclusion, increasing dietary MCFA levels up to 1.0% enhanced overall growth rate and feed intake, energy and protein digestibility in nursery pigs, improved energy status by lowering plasma β-hydroxybutyrate and free fatty acid levels, and reduced gut permeability in the early nursery period.

## Teaser

Increasing dietary MCFA levels up to 1.0% in early nursery diets (first 3 wk postweaning) enhanced postweaning growth rate, feed intake, energy and protein digestibility in nursery pigs and also improving energy status and gut barrier functions in the early nursery period.

## INTRODUCTION

The postweaning growth and feed intake is a key concern that significantly affects overall swine productivity as the feed consumption of newly weaned pigs is limited in the first week postweaning due to weaning stress, leading to a negative energy balance and body lipid catabolism (van Kempen et al., 2023). In addition, weaning stress, including dietary changes, can compromise gut health and digestive efficiency, which in turn negatively affects the pigs’ energy balance and hinders optimal growth and health (St-Pierre et al., 2023). Therefore, efficient energy sources are necessary to ensure sufficient energy supply and thereby improve the energy status and growth of piglets after weaning.

Medium-chain fatty acids (**MCFAs**) are saturated fatty acids with 6 to 12 carbon chain length that are known to have antibacterial and antiviral activities that can alleviate potential issues by pathogenic bacterial infection and postweaning diarrhea in weaning pigs (Cochrane et al., 2018; López-Colom et al., 2019). Although gut dysbiosis and postweaning diarrhea are critical issues in piglets during the weaning transition period, meeting energy needs is also important due to a negative energy balance. As the MCFAs consist of shorter chain fatty acids (C6-12) and do not necessarily require emulsification for digestion and absorption, it can be easily absorbed by the gut, preferentially transported via the portal vein to the liver instead of lymphatic vessels that transport long-chain fatty acids (C ≥ 14) and mainly undergo β-oxidation process for energy production (Odle, 1997; Zentek et al., 2011), while only a small portion of absorbed MCFA is stored or used for triglyceride resynthesis in adipose tissues (Odle, 1997). Thus, the MCFA supplementation in nursery diets can be beneficial for weaning pigs and be one of most effective ways to improve energy status in pigs after weaning compared with the other energy sources such as soybean oil.

Previous studies reported that increased dietary MCFA levels improved growth performance and feed efficiency in weaning pigs (Gebhardt et al., 2020; Kwon et al., 2025a). These studies indicate that the growth response of weaning pigs to increasing levels of MCFA may result from potential stabilization of gut microbiota under normal conditions and an improvement in energy supply (Gebhardt et al., 2020; Kwon et al., 2025a). However, there is limited information regarding its potential effects on energy status, gut permeability, and nutrient digestibility in nursery pigs, which need to be evaluated to utilize the MCFAs more effectively in nursery diets as an efficient and immediate source of energy for weaning pigs.

Therefore, the objective of this study was to evaluate the effect of increasing dietary MCFA levels in diets for newly weaned piglets on growth performance, fecal score, energy status by measuring blood β-hydroxybutyrate, and free fatty acids, gut permeability by measuring blood diamine oxidase and d-lactate levels, and apparent total tract digestibility (**ATTD**) of energy and nutrients.

## MATERIALS AND METHODS

### Animal Use Protocol

This experiment and sample collections were carried out in an environmentally controlled room at the University of Georgia Large Animal Research Unit (Winterville, GA). The experiment was conducted under protocols (#A2024 01-024-Y2-A0) approved by the Institutional Animal Care and Use Committee of the University of Georgia.

### Animals, Experimental Design, and Housing

A total of 100 pigs [Camborough × PIC337 and (Camborough × Berkshire) × PIC337; initial body weight (BW) of 5.72 ± 1.4 kg; weaned at 18.4 ± 1.8 d of age] were allotted to 5 treatments in 5 replicates with 4 pigs per pen (2 barrows and 2 gilts) based on BW in a randomized completely block design balanced with breed, sex, and littermate for a 35-d feeding trial. The treatments consisted of graded levels of MCFA at 0, 0.2, 0.5, 1.0, and 1.5%, replacing soybean oil. The MCFA product used was a free form of MCFA with a C6-12 MCFA blend from Fortiva (Vitacy^®^ S; Arden Hills, MN). The 2% of soybean oil was included in the 0.0% MCFA diet as a source of long-chain fatty acids and was replaced with the MCFA blend (*w/w*) for the other MCFA-supplemented diets from 0.2% to 1.5% levels to examine the effect of MCFA supplementation as an energy source in nursery pigs. The 1.5% MCFA treatment included 0.5% soybean oil to avoid potential negative impacts associated with the absence of long-chain fatty acid sources in nursery diets such as deficiencies of essential fatty acids (Jadhav and Annapure, 2023). All pigs were housed in nursery pens (1.0 m × 2.0 m) with woven-wire flooring and had free access to water and feed in an environmentally controlled nursery facility. No creep feed was provided during the lactation period. The two diet phases included d 0 to 21 postweaning (Phase 1) and d 21 to 35 postweaning (Phase 2). In Phase 1, the experimental diets were mixed with 0.3% Cr_2_O_3_ as an indigestible marker for ATTD measurements. In Phase 2, a common diet without MCFA supplementation was fed to all piglets to measure carry-over effects of MCFA supplementation in early nursery phase.

### Experimental Diets

All pigs were fed corn-soybean meal-based mash diets formulated to meet or exceed nutrient requirement estimates of NRC (2012) for pigs weighing 7 to 11 kg (d 0 to 21 postweaning) and 11 to 25 kg (d 21 to 35 postweaning; Table 1). A basal diet was first mixed without soybean oil to minimize variations in non-treatment diet components. This basal diet was then divided into five fractions. One fraction was mixed with 2% soybean oil without MCFA supplementation (the 0.0% MCFA diet). For the remaining fractions, soybean oil was replaced by the assigned MCFA levels of 0.2, 0.5, 1.0, and 1.5%, respectively, to create the MCFA-supplemented diets. Representative samples of mixed feed were collected at the feed mill and stored at −20 °C until analyzed for chemical analysis.

**Table 1.** Diet formulation and calculated chemical composition

Ingredient, %	Phase 1 (d 0 to 21 postweaning)	Phase 2^1^ (d 21 to 35 postweaning)
Corn	48.7635	54.2395
Soybean meal (dehulled)	20.00	32.50
Lactose	7.50	-
Dried distillers grains with solubles	5.00	7.50
Oat	5.00	-
Fish meal	3.75	-
HP300^2^	3.75	-
Soybean oil^3^	2.00	2.00
L-Lysine·HCl	0.617	0.446
L-Threonine	0.289	0.192
DL-Methionine	0.222	0.166
L-Valine	0.155	0.048
L-Tryptophan	0.082	0.048
L-Isoleucine	0.057	-
Vitamin mix^4^	0.20	0.20
Trace mineral mix^5^	0.15	0.15
Dicalcium phosphate	0.825	0.743
Limestone	0.984	1.140
Salt	0.620	0.615
Choline chloride 60%	0.023	-
Phytase^6^	0.0125	0.0125
Calculated chemical composition		
Metabolizable energy, kcal/kg	3,405	3,375
Crude protein, %	19.88	21.40
SID^7^ Lys, %	1.41	1.34
SID Met + Cys, %	0.79	0.79
SID Thr, %	0.90	0.86
SID Trp, %	0.27	0.25
Toal Ca, %	0.80	0.70
Available P^8^, %	0.53	0.43

^1^A common diet was fed to all pigs from d 21 to 35 postweaning.

^2^Hamlet Protein, Findlay, OH.

^3^Soybean oil was replaced by medium chain fatty acids at assigned levels.

^4^Vitamin premix supplied the following per kilogram of diet: 11,000 IU of vitamin A, 1,600 IU of vitamin D_3_, 99 IU of vitamin E, 4.4 mg of vitamin K, 55 µg of vitamin B_12_, 9.9 mg of riboflavin, 31.9 mg of pantothenic acid, 55 mg of niacin, 0.9 mg of folic acid, 3.9 mg of vitamin B_6_, 3.1 mg of thiamin, and 0.3 mg of biotin, 600 mg of choline chloride.

^5^Trace mineral premix supplied the following per kilogram of diet: 33 mg of Mn as manganous oxide, 110 mg of Fe as ferrous sulfate, 110 mg of Zn as zinc sulfate, 16.5 mg of Cu as copper sulfate, 0.3 mg of I as Ca iodate, 0.3 mg of Se as sodium selenite.

^6^625 FTU/kg diet (Quantum Blue 5G, AB Vista, Wiltshire, UK).

^7^SID = standardized ileal digestible.

^8^Available P from feed ingredients + P released by phytase.

### Data and Sample Collection

The pigs were individually weighed at the start of the trial (weaning), d 7, 14, 21, 28, and 35 post-weaning. The pen-based feed disappearance was measured when the pigs were weighed and average daily gain (**ADG**), average daily feed intake (**ADFI**), and gain-to-feed ratio (**G:F**) were calculated. The fecal score was recorded every day for the entire experimental period using a 4-scale fecal score system (1 = normal, 2 = soft, looser than normal feces, slight diarrhea, 3 = moderate diarrheic feces, and 4 = liquid, severe diarrhea) by observing individual pigs in each pen and assessing signs of stool consistency in the pen (Kwon et al., 2025a).

At d 7 and 21 postweaning, blood samples (10 mL) from 6 pigs (2 pigs per pen from the first replicate; 1 pig per pen from the remaining replicates) selected based on average BW were collected via jugular venipuncture in disposable vacutainer tubes containing the anticoagulant K_3_ EDTA (Becton Dickinson, Franklin, NJ). Plasma samples were obtained by centrifugation (Centra GP8R, Thermo IEC, MA), at 2,500 × g for 30 min at 4 °C and stored at –80 °C until analysis.

Fecal samples were collected from all pigs in each pen by rectal palpation at d 21 post-weaning, composited by pen, and stored at −20 °C until analysis. Then, the collected feces were thawed, dried in a forced-air drying oven at 55 °C for 5 d, ground through a 1-mm screen using a Wiley Laboratory Mill (Model 3; Arthur H. Thomas Co., Philadelphia, PA), and analyzed for gross energy (GE), dry matter (DM), crude protein (CP), ether extract (EE), ash, neutral detergent fiber (NDF), acid detergent fiber (ADF), and chromium for the ATTD, calculated according to Kong and Adeola (2014).

### Chemical Analysis

Feed and fecal samples in Phase 1 were analyzed for GE using a bomb calorimetry (Parr 6400 Automatic Isoperibol Bomb Calorimeter, Parr Instrument Company, IL), DM (AOAC, 2006; 934.01), CP (AOAC, 2006; 990.03), EE (AOAC, 2006; 920.39), ash (AOAC, 2006; 942.05), NDF (van Soest et al., 1991) and ADF (van Soest et al., 1991) at Agricultural Experimental Station Chemical Laboratories in the University of Missouri-Columbia (Columbia, MO; Table 2). Hemicellulose content was calculated by subtracting ADF content from NDF content. Chromium was analyzed at Feed and Environmental Water Laboratory in the University of Georgia. Briefly, 1 g of feed and feces samples were ashed on a muffle furnace at 550 °C for 6 hours followed by acid digestion of the ash according to AOAC (2006; 968.08). Then, the residue was quantitatively transferred to a digestion tube using two washes each with 5 mL of 25% HCl and digested in a pre-heated (125^o^C) digestion block (Martin Machine, Ivesdale, IL) for 30 min. The digests were cooled down to room temperature and diluted to a volume of 100 mL using deionized water and the solutions were analyzed for chromium following EPA Method 200.7 (USEPA, 1994) by Inductively Coupled Plasma-Optical Emission Spectroscopy (ICP-OES; Spectro Arcos FHS16, Germany).

**Table 2.** Postweaning growth performance of pigs fed diets with increasing MCFA levels over a 21-d period^1^

	MCFA level^2^, %						P-value	
Item	0.0	0.2	0.5	1.0	1.5	SEM	Linear	Quadratic
Body weight, kg								
d 0	5.69	5.67	5.66	5.74	5.80	0.61	0.25	0.60
d 7	6.19	6.24	6.31	6.36	6.15	0.09	0.91	0.02
d 14	8.50	8.49	9.03	8.79	8.69	0.16	0.20	0.02
d 21	11.19	11.12	11.90	11.68	11.77	0.33	0.04	0.18
d 28	15.87	15.67	16.95	16.74	16.76	0.36	0.01	0.10
d 35	19.90	20.10	21.17	21.41	21.34	0.447	0.01	0.10
Average daily gain, kg/d								
d 0 to 7	0.069	0.076	0.085	0.093	0.063	0.013	0.91	0.02
d 7 to 14	0.330	0.321	0.389	0.346	0.362	0.017	0.07	0.11
d 14 to 21	0.384	0.377	0.411	0.414	0.440	0.050	0.03	0.99
d 21 to 28	0.669	0.649	0.722	0.722	0.713	0.027	0.04	0.18
d 28 to 35	0.687	0.715	0.705	0.783	0.758	0.028	0.01	0.32
d 0 to 14	0.199	0.198	0.237	0.220	0.213	0.012	0.20	0.02
d 0 to 21 (Phase 1)	0.261	0.258	0.295	0.285	0.289	0.015	0.04	0.18
d 21 to 35 (Phase 2)	0.678	0.682	0.713	0.752	0.736	0.017	0.01	0.11
d 0 to 35 (Overall)	0.428	0.428	0.462	0.472	0.467	0.013	0.01	0.04
Average daily feed intake, kg/d								
d 0 to 7	0.141	0.158	0.162	0.167	0.151	0.010	0.45	0.05
d 7 to 14	0.419	0.404	0.485	0.421	0.420	0.021	0.94	0.08
d 14 to 21	0.628	0.611	0.668	0.660	0.703	0.040	0.01	0.91
d 21 to 28	0.956	0.940	1.033	1.007	1.008	0.057	0.22	0.34
d 28 to 35	1.038	1.077	1.176	1.196	1.174	0.057	0.03	0.11
d 0 to 14	0.291	0.288	0.334	0.300	0.294	0.016	0.92	0.08
d 0 to 21 (Phase 1)	0.403	0.395	0.445	0.420	0.431	0.016	0.12	0.28
d 21 to 35 (Phase 2)	0.997	1.008	1.104	1.102	1.091	0.052	0.04	0.12
d 0 to 35 (Overall)	0.641	0.640	0.709	0.693	0.695	0.028	0.04	0.13
G:F								
d 0 to 7	0.427	0.443	0.442	0.522	0.368	0.052	0.61	0.06
d 7 to 14	0.789	0.790	0.808	0.825	0.867	0.033	0.04	0.69
d 14 to 21	0.604	0.610	0.605	0.624	0.620	0.043	0.49	0.90
d 21 to 28	0.715	0.697	0.697	0.734	0.721	0.046	0.56	0.94
d 28 to 35	0.704	0.663	0.610	0.658	0.650	0.037	0.50	0.27
d 0 to 14	0.688	0.688	0.712	0.735	0.727	0.024	0.10	0.48
d 0 to 21 (Phase 1)	0.649	0.651	0.660	0.679	0.670	0.026	0.25	0.60
d 21 to 35 (Phase 2)	0.705	0.679	0.650	0.691	0.681	0.032	0.86	0.47
d 0 to 35 (Overall)	0.680	0.668	0.653	0.686	0.677	0.019	0.73	0.67

^1^N = 5 pens per treatment.

^2^Treatments: 0.0, 0.2, 0.5, 1.0, and 1.5% of medium-chain fatty acid (MCFA) levels in d 0 to 21 postweaning followed by feeding a common diet in d 21 to 35 postweaning. The MCFA was a blend of C6 to C12 in a free acid form. The 0.0% MCFA diet contained 2% soybean oil, which was replaced with MCFA at the assigned levels on a weight-to-weight basis.

Plasma samples were analyzed for β-hydroxybutyrate (colorimetric assay; Cayman Chemical Company, Ann Arbor, MI), free fatty acid (colorimetric assay; Zen-Bio, Inc., Durham, NC), diamine oxidase (ELISA; AFG Bioscience Northbrook, IL) and d-lactate (colorimetric assay; BioAssay Systems, Hayward, CA) using commercial assay kits following manufacturer’s instructions with a spectrophotometer (Multiskan Skyhigh, Thermo Fisher Scientific, Waltham, MA).

### Statistical Analysis

All data obtained in the current study were analyzed following a randomized complete block design using the PROC MIXED of SAS (ver. 9.4. SAS Inst. Inc., Cary, NC). A pen was used as an experimental unit to analyze growth performance, fecal score, and ATTD. An individual pig was used as an experimental unit for blood analyses. The models included the treatment as a fixed effect and the replicate as a random effect. The least-square means were separated using the PDIFF option in SAS. Orthogonal polynomial contrast analysis was performed to evaluate the linear and quadratic effects of increasing MCFA levels. The PROC IML was used to obtain coefficients for linear and quadratic analyses due to unequal spacing of treatments. Statistical differences were established at *P *≤ 0.05, and tendencies were established at 0.05 < *P* ≤ 0.10.

## RESULTS

With increasing MCFA levels, quadratic increases in BW (*P* ≤ 0.05) were observed at d 7 and 14 postweaning, followed by linear increases at d 21, 28, and 35 postweaning (*P* ≤ 0.05; Table 2), while there were also quadratic trends observed at d 28 and 35 postweaning (*P* = 0.10, tendency) with the greatest values at 0.5 and 1.0% MCFA levels, respectively. Average daily gain showed quadratic increases in d 0 to 7 and 0 to 14 postweaning (*P* ≤ 0.05) as the MCFA levels increased, with peak values at 1.0 and 0.5% MCFA levels, respectively, while ADG increased linearly in d 7 to 14 (*P* = 0.07, tendency), 14 to 21, 21 to 28, 28 to 35, 0 to 21 and 21 to 35 postweaning (*P* ≤ 0.05). Both linear and quadratic increases in ADG were observed in the overall period (d 0 to 35 postweaning; *P* ≤ 0.05) with a peak value at the 1.0% MCFA level. Average daily feed intake showed quadratic increases in d 0 to 7 (*P* ≤ 0.05), 7 to 14 (*P* = 0.08, tendency), 0 to 14 (*P *= 0.08, tendency) postweaning, as the MCFA levels increased, with peak values at 1.0%, 0.5%, and 0.5% MCFA levels, respectively. The ADFI linearly increased in d 14 to 21, 28 to 35, 21 to 35, and 0 to 35 postweaning (*P *≤ 0.05). The G:F ratio showed a quadratic increase in d 0 to 7 postweaning as the MCFA levels increased, with a peak value at the 1.0% MCFA level, while G:F increased linearly in d 7 to 14 (*P *≤ 0.05) and 0 to 14 (*P *= 0.10, tendency) postweaning with increasing dietary MCFA levels.

Fecal score increased linearly in d 21 to 28 and 21 to 35 postweaning (*P* ≤ 0.05; Table 3) with increasing dietary MCFA levels, while the fecal score was close to the lowest values in the entire experimental period.

**Table 3.** Postweaning fecal score of pigs fed diets with increasing MCFA levels over a 21-d period^1^

	MCFA level^2^, %						P-value	
Item	0.0	0.2	0.5	1.0	1.5	SEM	Linear	Quadratic
Fecal score^3^								
d 0 to 7	1.27	1.11	1.17	1.09	1.21	0.10	0.76	0.20
d 7 to 14	1.24	1.10	1.10	1.07	1.19	0.09	0.79	0.14
d 14 to 21	1.31	1.24	1.37	1.24	1.43	0.14	0.49	0.57
d 21 to 28	1.07	1.20	1.23	1.09	1.41	0.11	0.05	0.35
d 28 to 35	1.06	1.08	1.15	1.09	1.21	0.07	0.16	0.75
d 0 to 14	1.26	1.11	1.14	1.08	1.20	0.08	0.75	0.13
d 0 to 21 (Phase 1)	1.28	1.15	1.21	1.13	1.28	0.08	0.90	0.20
d 21 to 35 (Phase 2)	1.07	1.14	1.19	1.09	1.31	0.08	0.05	0.44
d 0 to 35 (Overall)	1.19	1.15	1.20	1.11	1.29	0.07	0.35	0.21

^1^N = 5 pens per treatment.

^2^Treatments: 0.0, 0.2, 0.5, 1.0, and 1.5% of medium-chain fatty acid (MCFA) levels in d 0 to 21 postweaning followed by feeding a common diet in d 21 to 35 postweaning. The MCFA was a blend of C6 to C12 in a free acid form. The 0.0% MCFA diet contained 2% soybean oil, which was replaced with MCFA at the assigned levels on a weight-to-weight basis.

^3^Fecal score was measured using a 4-scale fecal score system (1 = normal, 2 = soft, looser than normal feces, slight diarrhea, 3 = moderate diarrheic feces, and 4 = liquid, severe diarrhea) by observing individual pigs in each pen and assessing signs of stool consistency in the pen.

The ATTD of GE (*P* ≤ 0.05), DM (*P* = 0.10, tendency) and CP (*P* = 0.07, tendency) increased linearly with increasing MCFA levels (Table 4), although there were no significant differences in the ATTD of EE, ash, NDF, ADF and hemicellulose.

**Table 4.** Apparent total tract digestibility of weaning pigs fed diets with increasing MCFA levels over a 21-d period^1^

	MCFA level^2^, %						P-value	
Item	0.0	0.2	0.5	1.0	1.5	SEM	Linear	Quadratic
Gross energy, %	73.06	73.77	71.85	76.95	75.48	1.73	0.04	0.91
Dry matter, %	75.73	76.49	74.66	78.94	77.22	1.33	0.10	0.75
Crude protein, %	65.79	67.12	64.23	71.57	69.19	2.37	0.07	0.82
Ether extract, %	35.14	40.56	33.10	43.84	39.44	7.80	0.40	0.78
Ash, %	55.19	54.14	48.12	58.40	50.12	3.24	0.51	0.77
Neutral detergent fiber, %	45.89	43.05	42.06	49.96	45.83	2.65	0.36	0.96
Acid detergent fiber, %	45.39	43.84	43.42	50.09	46.32	2.74	0.33	0.75
Hemicellulose, %	46.14	42.66	41.38	49.90	45.56	2.74	0.39	0.93

^1^N = 5 pens per treatment at d 21 postweaning.

^2^Treatments: 0.0, 0.2, 0.5, 1.0, and 1.5% of medium-chain fatty acid (MCFA) levels in d 0 to 21 postweaning followed by feeding a common diet in d 21 to 35 postweaning. The MCFA was a blend of C6 to C12 in a free acid form. The 0.0% MCFA diet contained 2% soybean oil, which was replaced with MCFA at the assigned levels on a weight-to-weight basis.

There were no significant differences in plasma β-hydroxybutyrate and free fatty acid levels at d 21 postweaning (Table 5). Plasma β-hydroxybutyrate levels decreased linearly (*P* = 0.06, tendency) at d 7 postweaning, while plasma free fatty acid levels showed linear (*P *≤ 0.05) and quadratic (*P* = 0.06, tendency) responses as the MCFA levels increased, with the lowest value at 0.5% MCFA level.

**Table 5.** Plasma β-hydroxybutyrate and free fatty acid levels in weaning pigs fed diets with increasing MCFA levels over a 21-d period^1^

	MCFA level^2^, %						P-value	
Item	0.0	0.2	0.5	1.0	1.5	SEM	Linear	Quadratic
β-hydroxybutyrate, µM								
d 7 postweaning	160.94	156.02	137.86	152.25	138.20	8.26	0.06	0.53
d 21 postweaning	111.19	104.82	105.10	105.07	105.13	3.61	0.40	0.40
Free fatty acid, µM								
d 7 postweaning	159.17	155.90	142.73	177.33	222.81	24.26	0.01	0.06
d 21 postweaning	80.53	82.42	80.94	81.07	80.98	7.64	0.97	0.97

^1^N = 6 pigs per treatment.

^2^Treatments: 0.0, 0.2, 0.5, 1.0, and 1.5% of medium-chain fatty acid (MCFA) levels in d 0 to 21 postweaning followed by feeding a common diet in d 21 to 35 postweaning. The MCFA was a blend of C6 to C12 in a free acid form. The 0.0% MCFA diet contained 2% soybean oil, which was replaced with MCFA at the assigned levels on a weight-to-weight basis.

Plasma diamine oxidase levels tended to decrease linearly with increasing dietary MCFA levels at d 7 postweaning (*P* = 0.06, tendency; Table 6), while there was no significant difference at d 21 postweaning. Plasma d-lactate levels showed quadratic responses at both d 7 (quadratic, *P* ≤ 0.05; linear, *P *= 0.07) and 21 (*P* = 0.10, tendency) postweaning, with lower values observed in dietary MCFA levels ranging from 0.2 to 1.0% compared to the 0.0% MCFA level, while the 1.5% MCFA level had the greatest values among dietary treatments.

**Table 6.** Plasma diamine oxidase and d-lactate levels in weaning pigs fed diets with increasing MCFA levels over a 21-d period^1^

	MCFA level^2^, %						P-value	
Item	0.0	0.2	0.5	1.0	1.5	SEM	Linear	Quadratic
Diamine oxidase, ng/mL								
d 7 postweaning	25.40	24.84	24.87	19.31	14.57	6.53	0.06	0.65
d 21 postweaning	32.52	33.29	34.14	31.60	33.87	2.09	0.90	0.85
D-lactate, mM								
d 7 postweaning	0.685	0.592	0.626	0.622	0.813	0.072	0.07	0.05
d 21 postweaning	0.296	0.166	0.179	0.232	0.348	0.079	0.28	0.10

^1^N = 6 pigs per treatment.

^2^Treatments: 0.0, 0.2, 0.5, 1.0, and 1.5% of medium-chain fatty acid (MCFA) levels in d 0 to 21 postweaning followed by feeding a common diet in d 21 to 35 postweaning. The MCFA was a blend of C6 to C12 in a free acid form. The 0.0% MCFA diet contained 2% soybean oil, which was replaced with MCFA at the assigned levels on a weight-to-weight basis.

## DISCUSSION

MCFAs can also be effective and instant energy sources, which can reduce body lipid catabolism caused by a negative energy balance in the weaning transition period (van Kempen et al., 2023), due to its unique characteristics including faster digestion, absorption, and utilization as an energy source, rather than being stored in the body like long-chain fatty acids. In addition, MCFAs are known to have antibacterial and antiviral properties, which can be utilized in nursery pig diets to help mitigate issues associated with weaning such as gut dysbiosis and postweaning diarrhea (Zentek et al., 2011). Therefore, this study evaluated the effect of increasing MCFA levels in early nursery pig diets during the first 3 wk postweaning on growth performance, fecal score, nutrient digestibility, energy status, and gut permeability in pigs.

In the current study, increasing MCFA levels quadratically increased pig BW and growth rate in early nursery period (up to d 14 postweaning) with the greatest values observed at the 0.5% to 1.0% of MCFA levels. These results are likely attributed to similar quadratic increases observed in feed intake and feed efficiency during the same period, indicating that MCFA supplementation up to 1.0% can improve early postweaning growth performance, which agreed with Gebhardt et al. (2020) who reported that MCFA supplementation up to 1.5% improved growth rate linearly throughout the nursery period. In the current study, dietary MCFA levels up to 1.0% resulted in reductions in blood β-hydroxybutyrate and free fatty acid levels at d 7 postweaning, indicating improved negative energy balance (Perri et al., 2016; Ren et al., 2017) as well as decreased in blood diamine oxidase and d-lactate levels at d 7 postweaning, suggesting reduced gut permeability (Engelsmann et al., 2023). These results could contribute to this improved growth performance. However, in the current study, a slightly lower growth rate, feed intake and efficiency were observed at 1.5% of MCFA level compared to lower MCFA levels. This may be attributed to slight increases in plasma d-lactate and free fatty acid levels, indicating increased gut permeability and a negative energy balance. In addition, the high level of MCFA in the free form may have negatively impacted feed intake in nursery pigs, potentially due to increased acidity, strong odor, and influence on satiety signaling (Mabayo et al., 1992; Zentek et al., 2011). Cera et al. (1989) reported that supplementation of free-form MCFA at 8% reduced feed intake in weaning pigs. Therefore, these results indicate that MCFA supplementation up to 1.0% can be beneficial for early postweaning performance, while higher levels of MCFA may not further improve feed intake or growth rate, although overall performance may be enhanced with extended feeding.

However, Gebhardt et al. (2020) reported a linear increase in feed intake and growth rate when the free form of MCFA (a 1:1:1 blend of C6, C8 and C10) was supplemented to nursery diets up to 1.5%, at the expense of soybean oil. In addition, our previous study (Kwon et al., 2025a) reported that increasing MCFA levels up to 1.6% in the form of free fatty acids (a 1:1 blend of C6 and C8) at the expense of soybean oil linearly improved growth rate and feed efficiency in d 7 to 21 postweaning, with no effect in feed intake. Although these previous studies also used the free form of MCFA, replacing soybean oil, their composition differed from that used in the current study, which included a broader blend of C6 to C12 in the form of free fatty acids. Each MCFA from C6 to 12 exhibits varying antibacterial effects against *E. coli* and *Salmonella* in the intestinal porcine epithelial cell line (Martínez-Vallespín et al., 2016). These MCFAs also differ in their energy potentials (Lin et al., 2021), which may contribute to differences in the efficacy of MCFA on growth rate and feed intake of pigs.

After 14 d postweaning, BW and growth rate increased linearly with increasing MCFA levels until the end of the trial (d 35 postweaning), while the greatest values were still observed at the MCFA levels of 0.5% to 1.0%, despite all pigs being fed a common diet from d 21 to 35 postweaning. Similarly, postweaning feed intake from d 14 to 35 postweaning increased linearly with increasing MCFA levels, while no differences were observed in feed efficiency. These results agreed with Kwon et al. (2025a) who reported that increasing MCFA levels up to 1.6% for nursery pigs during 28 d after weaning linearly increased pig BW and growth rate in the late nursery period (d 14 to 28 postweaning). In the current study, there were linear increases in the ATTD of GE, DM and CP at d 21 postweaning with increasing MCFA levels, peaking at the 1.0% of MCFA level, which are consistent with the growth performance results observed. These results are consistent with Hong et al. (2012) who reported that MCFA supplementation improved DM, CP, and energy digestibility in weaning pigs. MCFA supplementation has been reported to restore villus height and crypt depth following LPS-challenges (De Keyser et al., 2019) and enhance digestive enzyme activities of weaning pigs (Cui et al., 2022), potentially improving digestive and absorptive capacities. Thus, enhanced growth performance observed with MCFA supplementation in nursery pigs is likely due to improvements in digestive and absorptive capacities in pigs as evidenced in enhanced energy and protein digestibility. In addition, these results suggest a carry-over effect of MCFA supplementation as pigs over 0.5% of MCFA supplementation groups still maintained greater growth rate and feed intake than those in the 0.0% of MCFA group during the common diet phase. This can be due to enhanced growth performance and energy and protein digestibility in the early nursery period, along with improved energy status evidenced by reduced blood β-hydroxybutyrate, free fatty acid and improved gut barrier function evidenced by decreased blood diamine oxidase and d-lactate levels at d 7 postweaning. These benefits observed in the current study likely continued to support growth performance even after all pigs were fed a common diet following the period of feeding MCFA-supplemented diets. However, as no further improvement in growth performance or the ATTD of GE, DM and CP were observed at the MCFA levels above 1.0%, the optimal supplementation level based on the findings of the current study appears to be between 0.5 and 1.0%.

In the current study, fecal score increased linearly with increasing MCFA levels in the common diet phase (d 21 to 35 postweaning). Kwon et al. (2025a) reported a reduction in fecal scores with increasing MCFA levels up to 1.6% in the first 3 wk of the nursery period. The antibacterial properties of MCFA, along with their ability to reduce intestinal pH as a free fatty acid form, contribute to the development of a healthier gut environment (Zentek et al., 2013; López-Colom et al., 2019; Matsui et al., 2021), which can improve fecal consistency (Kwon et al., 2025a). Although the fecal score increased with increasing MCFA levels in the common diet phase (d 21 to 35 postweaning) of the current study after feeding MCFA-supplemented diets in d 0 to 21 postweaning, the overall fecal score remained low, indicating the absence of severe diarrhea. This may have limited the detection of a clear effect of MCFA on fecal consistency and is likely due to the use of a relatively low protein (20%) diet, as low protein diets have been shown to reduce the occurrence of postweaning diarrhea (Lawal et al., 2024).

Plasma β-hydroxybutyrate levels decreased linearly at d 7 postweaning, while plasma free fatty acid levels showed both linear and quadratic responses as MCFA supplementation levels increased, with the lowest values observed at the 0.5% MCFA level, which corresponded to the greatest growth performance in the early nursery period. These results agree with the previous studies reporting MCFA supplementation reduced plasma β-hydroxybutyrate and free fatty acid levels in weaning pigs (Diether et al., 2023; Kwon et al., 2025a). Plasma β-hydroxybutyrate and free fatty acid concentrations are commonly used as indicators of body lipid catabolism, with elevated plasma levels reflecting increased mobilization and oxidation of lipid stores during energy-deficient states (Perri et al., 2016; Ren et al., 2017). MCFAs are considered ketogenic due to their rapid absorption and hepatic oxidation, which under conditions of negative energy balance such as fasting or low blood glucose promotes the production of ketone bodies like β-hydroxybutyrate (Zentek et al., 2011). However, in the current study, MCFA supplementation resulted in a reduction in plasma β-hydroxybutyrate levels at d 7 postweaning, suggesting that the piglets fed the MCFA-supplemented diets were likely not under significant negative energy balance, as shown by decreased plasma free fatty acid levels with increasing dietary MCFA levels up to 0.5%. It is also possible that MCFAs were efficiently utilized for energy production via the β-oxidation, thereby limiting their availability for conversion into ketone bodies (Schönfeld and Wojtczak, 2016). Therefore, these results indicate that supplementing nursery diets with MCFAs up to 0.5% has potential to mitigate negative energy balance in the early nursery period. However, plasma free fatty acid levels increased at 1.0 and 1.5% of MCFA levels over 0.0% of MCFA level at d 7 postweaning. This result contrasts with Kwon et al. (2025a) who reported linear decreases in plasma free fatty acid levels at d 14 postweaning with increasing MCFA levels up to 1.6%. The current study measured these parameters at d 7 postweaning, when pigs may experience greater weaning stress and negative energy balance due to low feed intake compared with d 14 postweaning (van Kempen et al., 2023). In addition, feed intake in d 0 to 7 postweaning showed a quadratic response, with lower feed intake and feed efficiency at the 1.5% MCFA level compared to other MCFA levels. This reduction in feed, and consequently energy intake, could potentially lead to increased body lipid catabolism and elevated free fatty acid production, as plasma free fatty acid levels are known to increase during energy-deficient states, such as fasting or anorexia, reflecting increased mobilization and oxidation of lipid stores (Perri et al., 2016; Ren et al., 2017). However, since energy status varies with feed intake at different nursery stages, further research is needed to evaluate how energy status markers respond to varying MCFA supplementation levels throughout these stages.

There were no significant differences in plasma β-hydroxybutyrate and free fatty acid levels at d 21 postweaning, which is consistent with Kwon et al. (2025a) reporting no difference in plasma free fatty acid levels at d 28 postweaning with increasing MCFA levels up to 1.5%. These results suggest that although MCFA was supplemented to nursery diets only in d 0 to 21 postweaning, the effect of MCFA becomes less effective at supporting body lipid catabolism caused by negative energy balance, compared to the early nursery period (d 7 postweaning), likely because feed intake increases as the nursery period progresses although its supplementation can still enhance growth performance in the late nursery period (d 21 to 35 postweaning) with feeding a common diet for all pigs.

Plasma diamine oxidase levels tended to decrease linearly with increasing MCFA levels at d 7 postweaning, although no significant differences were observed at d 21 postweaning. Plasma d-lactate levels showed quadratic responses on both d 7 and 21 postweaning, with lower values detected at MCFA supplementation levels between 0.2 and 1.0% compared to the 0.0% of MCFA level. These blood markers have been widely used to measure gut permeability in weaning pigs, with greater levels indicating gut barrier dysfunction or leaky gut (Tossou et al., 2016; Ding et al., 2023; Kwon et al., 2025b). Lee and Kang (2017) reported that the MCFA treatment enhanced the mRNA gene expression of tight junction proteins such as Zonula Occludens-1 and Occuldin in IPEC-J2 cells and pigs challenged by cholera toxin when 0.5% capric acid (C10:0) was supplemented to diets, while reducing that of proinflammatory cytokines. This can be due to the properties of MCFA, which can modulate gut microbiota and exert antibacterial activity against pathogenic bacteria. These actions help reduce the release of bacterial toxin, oxidative stress, and inflammation in the gut, ultimately contributing to reduced gut permeability and improved postweaning growth performance (Zentek et al., 2013; Lee and Kang, 2017). Interestingly, an increase in plasma d-lactate levels was observed when MCFA supplementation levels increased from 1.0% to 1.5% at d 7 and 21 postweaning. This result may be related to the growth performance that did not show any benefit at the 1.5% of MCFA level compared with the lower MCFA levels. Plasma free fatty acid levels also increased at the 1.0% to 1.5% MCFA supplementation levels compared to the 0.2% to 0.5% at d 7 postweaning. Increased gut permeability has been associated with the translocation of toxins, bacteria, and dietary antigens across the intestinal epithelial barrier, potentially triggering inflammation, reducing feed intake reduction and impairing growth performance (Campbell et al., 2013). Although the mechanisms underlying increased plasma d-lactate and free fatty acid levels observed with high MCFA levels are not yet fully understood, supplementation with high MCFA levels (e.g., 1.5%) may be ineffective in alleviating negative energy balance and could potentially exacerbate gut permeability, thereby resulting in no additional performance benefit over lower supplementation levels in the early nursery period. Therefore, further studies are needed to demonstrate how excessive MCFA levels might limit their own beneficial effects on gut permeability and energy status compared with lower supplementation levels in the weaning transition period.

In conclusion, increasing dietary MCFA levels increased the overall growth rate, feed intake, energy and protein digestibility of nursery pigs, with optimal responses observed up to the 1.0% level and a carry-over effect was observed in late nursery period. MCFA supplementation also improved pig body energy status by decreasing β-hydroxybutyrate and free fatty acids in the first week of weaning when the 0.5% of MCFA was supplemented to the diets and reduced gut permeability when up to 1.0% of MCFA was supplemented. Therefore, up to 1.0% of MCFA supplementation for weaning pigs can be beneficial to enhance postweaning growth performance, while improving energy status and gut barrier function. These beneficial effects of MCFA supplementation can be maintained in the late nursery period after feeding MCFA-containing diets in the first 3 wk of nursery period.
